# Twist, or Rotation of the Colon

**Published:** 1898-07

**Authors:** Thomas V. Simpson

**Affiliations:** Yorktown, N. W. T., Canada


### TWIST, OR ROTATION OF THE COLON.

By Thomas V. Simpson, V.S.,
YORKTOWN, N. W. T., CANADA.

*Subject.* Gelding, three years old past, driver, weight about 900 pounds.

*History.* Was taken sick during the night, and on the following morning when first seen presented the following symptoms: Subacute abdominal pain; would lie down a great deal; passed a very small quantity of hard feces. Seemed very dull, and rubbed tail against side of stall. The pulse was very little quickened, and no abnormal rise of temperature. Rectal examination showed no impaction of colon.

*Treatment.* Gave one ounce of aloes and two drachms of powdered ginger in solution, thrown back on the tongue with a syringe. In the afternoon grew worse, and no feces having been passed, I gave barium chloride, seven grains, intravenously, after which some hard feces were passed, assisted by the administration of rectal injections of warm water. At night he seemed to be getting worse, and as the bowels had not moved to any extent I gave ten grains more of barium chloride intravenously, with the result of securing the passage of only a very small quantity of fecal matter. At this time the pulse was quick and almost imperceptible, temperature 103°, pain acute and constant, would paw and lie down, and assume the dorsal position when down. Seeing it was impossible to get a cathartic to operate, I gave chloral hydrate, which eased the pain and kept the patient quiet. Death followed at 2 P.M. the succeeding day. Before succumbing the pains became very acute; animal would roll and assume dorsal position; pulse again imperceptible at jaw; abdomen tucked; would quiver and sweat.

*Postmortem.* Examination of abdominal organs revealed a volvulus, the large colon being twisted at the sternal and diaphragmatic flexures. Very little food was found in the alimentary canal. This case answers to the condition above named as described in Möller's *Surgery.*

